# Compound-Specific and Intramolecular δ^15^N Analysis of a Poly-Nitrogenous Amino Acid: Histidine

**DOI:** 10.1021/acs.analchem.5c01711

**Published:** 2025-07-24

**Authors:** Charlotte Wing Man Lee, Mark A. Altabet, Jesus Baca, Jason Barrera, Lin Zhang

**Affiliations:** † 14738 Texas A&M University Corpus Christi, Corpus Christi, Texas 78412, United States; ‡ School for Marine Science and Technology, University of Massachusetts Dartmouth, New Bedford, Massachusetts 02744, United States

## Abstract

Histidine (HIS) is an essential amino acid (AA) with key physiological roles in metal chelation and proton buffering. Its three nitrogen (N) atoms—one α-amino and two in the imidazole side chain—are incorporated through distinct biosynthetic pathways and undergo different catabolic processes. Thus, its intramolecular δ^15^N values likely provide additional information on these pathways and associated N fluxes. Very few studies have reported molecular average δ^15^N_HIS_ (δ^15^N_HIS‑Total_) values, and there are no reported intramolecular δ^15^N_HIS_ data for natural materials due to technical limitations of available methods. Here, we present a novel analytical approach for compound-specific and intramolecular δ^15^N values of poly-nitrogenous AAs using HIS as an example. This scheme can be adapted to obtain position-specific δ^15^N values of other poly-nitrogenous AAs such as glutamine. Underivatized HIS is separated by ion-exchange chromatography (IC) and divided into two aliquots. One fraction is fully oxidized to NO_3_^–^ using UV-persulfate oxidation for δ^15^N_HIS‑Total_ measurement, while the other undergoes NaClO oxidation, selectively converting α-N and a minor fraction of side chain-N to NO_2_^–^ at a known ratio. The δ^15^N_HIS_ values of α-N (δ^15^N_HIS‑α_) and side chain-N (δ^15^N_HIS‑s_) are then calculated from these two results. Our findings reveal that α-N is consistently enriched in ^15^N relative to side chain-N in both commercial HIS powder (Δδ^15^N_α‑s_ = ∼ +8‰) and biological samples (Δδ^15^N_α‑s_ = ∼+3 to 25‰), likely due to preferential α-N catabolism via deamination. This finding supports the potential of studying diverse biosynthetic and catabolic processes of poly-nitrogenous AAs using intramolecular N isotope analysis.

## Graphical abstract

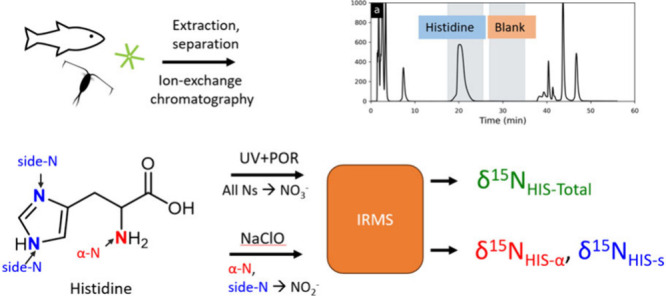

## Introduction

Nitrogen (N) isotopic composition (δ^15^N) of amino acids (AAs) has been widely applied to study N assimilation, biosynthesis, and metabolism, as well as trophic dynamics in ecosystems.^1–5^ The interpretative power of this tool stems from the N isotopic fractionation induced by biochemical reactions involving the breakage or formation of C–N bonds, mostly driven by transamination and deamination of the α-N.^6,7^ However, the intramolecular ^15^N distribution within poly-nitrogenous AAs, where side chain-N atoms may experience different biochemical reactions compared to the α-N, remains largely unexplored.

Histidine (HIS) is a structurally unique essential amino acid containing three nitrogen atoms. During HIS biosynthesis in primary producers, the α-N is incorporated via transamination from glutamic acid, while the two N atoms in the imidazole side chain originate from glutamine and ATP, respectively^8,9^ (Figure SF1a). Some AAs such as glutamic acid, aspartic acid, valine, alanine, leucine, and isoleucine readily exchange N with each other and with cellular ammonia through transamination—collectively referred to as the “metabolic N pool”.^10^ HIS does not undergo significant N exchange with this pool in vivo.^11,12^ As a result, the molecular average δ^15^N value of HIS (δ^15^N_HIS‑Total_) is decoupled from those of these AAs,^13–16^ which are typically significantly enriched in ^15^N with increasing trophic levels.^10^ Given the high biosynthetic cost of HIS (∼41 ATP),^8,17^ organisms may favor direct assimilation of dietary HIS over de novo synthesis or catabolism, thus preserving the original δ^15^N_HIS‑Total_ value of dietary sources.^18^ Consequently, δ^15^N_HIS‑Total_ has been suggested as an indicator of the δ^15^N composition of the basal N source in an ecosystem.^10^

In addition to δ^15^N_HIS‑Total_ values, intramolecular δ^15^N_HIS_ values could provide insight into biosynthetic pathways and the corresponding isotope fractionation that each N atom experiences. The α-N can be catabolized via irreversible deamination by histidine ammonia-lyase (Figure SF1b), whereas complete degradation pathways for the imidazole ring are absent in many lower eukaryotes and plants.^19,20^ Compared to the side chain-N atoms, the α-N atom is more likely subjected to HIS catabolism that irreversibly cleaves the C–N bond and thus induces isotopic fractionation to the α-N atom. The two N atoms in the imidazole ring might preserve the original isotopic composition of HIS imprinted from biosynthesis by primary producers with minimal alteration. As a result, the δ^15^N value of side chain-N (δ^15^N_HIS‑s_) may retain the original biosynthetic isotope signals, while the δ^15^N value of α-N (δ^15^N_HIS‑α_) may reflect the isotopic fractionation through deamination, which is not directly related to the ^15^N enrichment in the metabolic N pool caused by transamination. These differences could provide insights into physiological and nutritional status, independent of trophic fractionation effects.

Despite its potential, δ^15^N_HIS_ measurements have been limited by analytical challenges. Traditional δ^15^N_HIS_ analysis using gas chromatography-combustion-isotope ratio mass spectrometry (GC/C/IRMS) requires derivatization. Common HIS derivatives exhibit low recovery, long retention times, and frequent coelution with other AAs.^12,21–25^ The only reported attempt to measure intramolecular δ^15^N_HIS_ values was by Sacks and Brenna,^26^ who enzymatically cleaved the α-N and analyzed the δ^15^N values of HIS and the cleaved product via GC/C/IRMS, but no intramolecular δ^15^N_HIS_ data for natural materials exist in the literature. Offline AA purification using liquid chromatography coupled with elemental analyzer-IRMS (EA-IRMS) provides an alternative method by eliminating the need for derivatization. Underivatized AAs are separated and fraction-collected via high-performance liquid chromatography (HPLC) and analyzed for δ^15^N values using EA-IRMS.^27^ To the best of our knowledge, only one study has reported δ^15^N_HIS_ data of natural samples analyzed using this HPLC/EA-IRMS approach.^28^

To address these limitations, we present a novel analytical framework for compound-specific and intramolecular δ^15^N-AA analysis for poly-nitrogenous AAs using HIS as a model. The method integrates ion-exchange chromatography (IC) purification, UV-persulfate oxidation (UV+POR), NaClO oxidation, and purge-and-trap IRMS to determine the values of δ^15^N_HIS‑Total,_ δ^15^N_HIS‑α_, and δ^15^N_HIS‑s_. Validation with seven in-house HIS isotopic standards and five biological samples demonstrated high accuracy and precision. This approach enables new applications of intramolecular δ^15^N-AA analysis to explore N metabolism and biosynthetic pathways.

## Experimental Section

### Standard Preparation

A working standard solution of histidine (HIS-0) was prepared from a commercial l-histidine powder (VWR, PA, USA) without isotopic enrichment. To generate HIS calibration standards with a wide range of δ^15^N values, HIS-0 was mixed gravimetrically with isotopically labeled l-histidine monohydrochloride monohydrate standards (Cambridge Isotope, MA, USA) at known proportions. The *α-series* (HIS-α1, α2, α3) was produced by mixing HIS-0 with a labeled l-histidine standard enriched in ^15^N exclusively at the α-position (98% ^15^N), while the *w-series* (HIS-w1, w2, w3) was prepared using an l-histidine standard uniformly labeled with 98% ^15^N at all N positions. The molecular average δ^15^N value of each standard (δ^15^N_HIS‑Total_) was determined by elemental analyzer-isotope ratio mass spectrometry (EA-IRMS) at the University of California Davis Stable Isotope Facility (Table 1). Additionally, a urocanic acid solution was prepared from a commercial powder (Acros, USA) as a structural analog of the HIS imidazole side chain. This urocanic acid standard was used to evaluate the reactivity and isotopic behavior of side chain-N during subsequent oxidation steps.

**Table 1:** δ^15^N Values of α-N and Side Chain-N of HIS Isotopic Standards Calculated from δ^15^N_ClO_ Values of IC-Collected HIS Fractions and δ^15^N_HIS‑Total_ Values Determined by EA-IRMS

Standard	δ^15^N_HIS‑Total_ (‰)	δ^15^N_ClO_ (‰)	δ^15^N_HIS‑s_ (‰)	δ^15^N_HIS‑α_ (‰)
His-0	–6.0 ± 0.0	–1.8 ± 0.6	–8.6 ± 0.5	–0.7 ± 0.5
His-a1	1.2 ± 0.0	16.2 ± 0.6	–8.7 ± 0.6	20.9 ± 0.6
His-a2	8.2 ± 0.1	32.4 ± 0.3	–7.9 ± 0.5	40.3 ± 0.5
His-a3	15.3 ± 0.0	51.5 ± 1.3	–8.8 ± 1.1	63.5 ± 1.1
His-w1	10.4 ± 0.0	12.8 ± 0.6	9.0 ± 0.5	13.2 ± 0.5
His-w2	26.7 ± 0.1	28.6 ± 0.1	25.6 ± 0.4	28.8 ± 0.4
His-w3	43.1 ± 0.0	49.4 ± 0.2	39.2 ± 0.4	51.1 ± 0.4

To evaluate HIS separation from other AAs, seven standard AA mixtures were prepared by combining each HIS isotopic standard (HIS-0, -α1, -α2, -α3, -w1, -w2, -w3) with l-alanine (Ala), l-arginine (Arg), l-asparagine (Asn), d/l-aspartic acid (Asp), l-cysteine (Cys), l-glutamic acid (Glu), l-glutamine (Gln), glycine (Gly), l-isoleucine (Ile), l-leucine (Leu), l-lysine (Lys), d/l-methionine (Met), l-phenylalanine (Phe), d/l-serine (Ser), d/l-threonine (Thr), l-tyrosine (Tyr), and d/l-valine (Val). The final AA concentrations were adjusted to 5 mM for HIS, Asp, Glu, Gln, and Phe, and 2.5 mM for all other AAs.

### Sample Preparation

To further validate the method for natural samples, a suite of materials was analyzed, including: 1) a cyanobacteria powder (*Spirulina Pacifica*) that has been used as a multilab quality control standard for different compound-specific isotope analysis of AA (CSIA-AA) methods.^29,30^ 2) Copepod and euphausiid samples that were collected from the eastern tropical North Pacific (ETNP; 14.02°N, 104.27°W) during the R/V *Sally Ride* (*SR2011*, Scripps Institution of Oceanography) cruise from December 2020 to January 2021.^31^ 3) Particulate organic matter (POM) in the size fraction of 0.3 – 53 μm that was collected using a precombusted glass fiber filter (GF-75, 0.3 μm pore size) during the same cruise at 35 m depth using a McLane large-volume in situ filtration system (WTS-LV; McLane Research Laboratories).^32^ 4) A muscle tissue sample from a striped mullet (*Mugil cephalus*) that was collected in Nueces Bay, TX, in 2014. Sample collection details are provided in Table ST1.

Extraction of AAs including HIS from natural samples followed previously established protocols (Figure 1).^29,30,33,34^ Briefly, 15–50 mg of dried biological samples or three-quarters of a GF-75 filter were hydrolyzed in 6 N HCl at 110 °C for 22 h to release AAs from proteins. This sample mass was chosen to ensure sufficient recovery of HIS, given its relatively low abundance in natural materials. Following hydrolysis, lipids and metal ions were removed from the samples by washing with *n*-hexane/dichloromethane (6:5, v/v) and cation-exchange chromatography respectively following previously established protocols.^30,35^ The 10% NH_4_OH fraction containing HIS and other AAs was dried at 35 °C under vacuum and redissolved in 0.2–7 mL of Milli-Q water to achieve a target HIS concentration of 2–5 mM before chromatographic separation.

**Figure 1:**
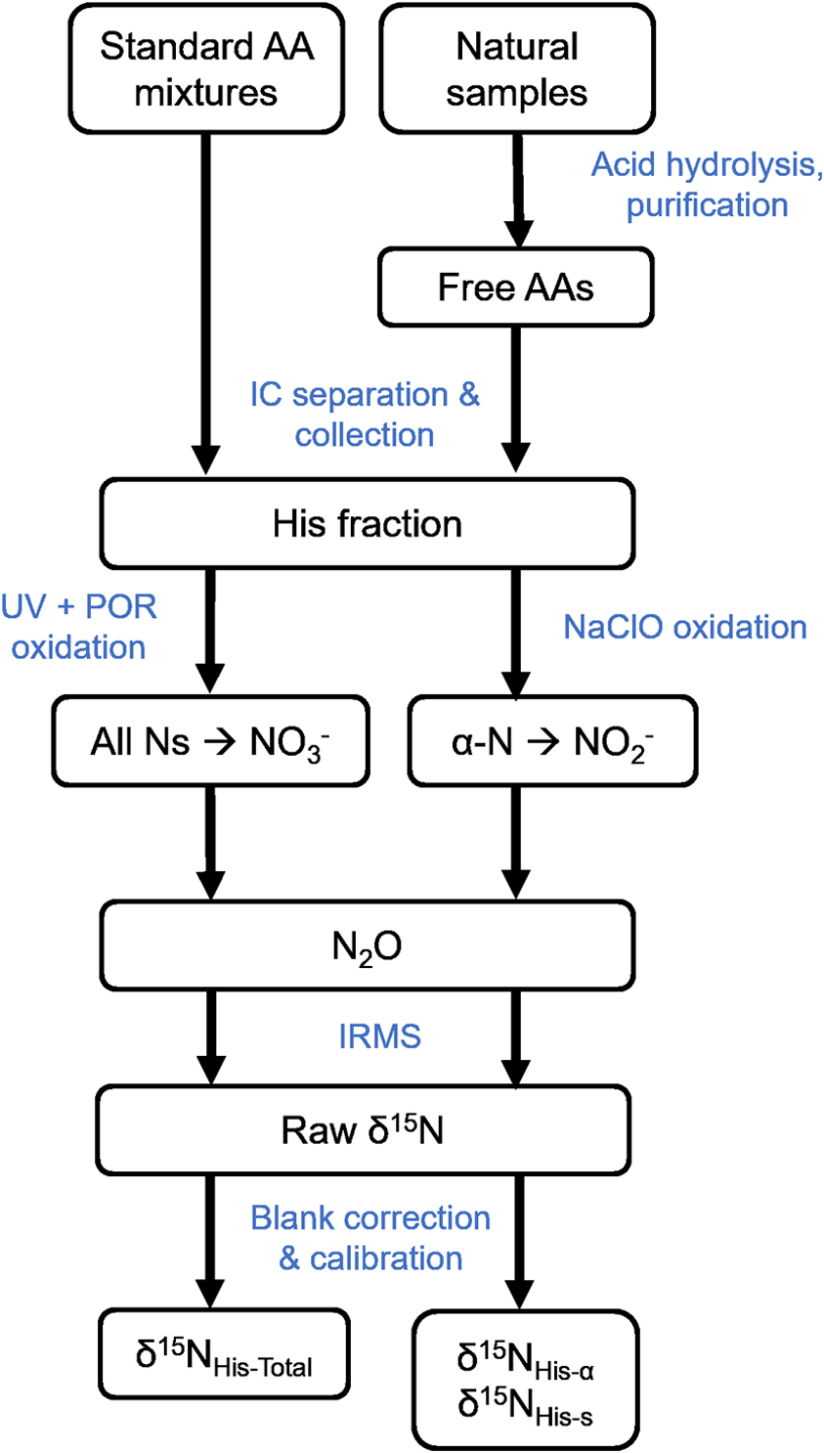
Procedural flowchart of δ^15^N_HIS_ analysis of standards and biological samples.

### Ion-Exchange Chromatography Separation and Collection

HIS was isolated from standard mixtures and natural samples using an ion-exchange chromatography system (ICS 5000+; Thermo Fisher Scientific) equipped with a CarboPac PA10 semipreparative column (9 × 250 mm, 10 μm particle size, < 10 Å pore size). An automated fraction collector (AFC 3000; Thermo Fisher Scientific) received ∼90% of the flow, while the rest was directed to a pulse amperometric detector (Thermo Fisher Scientific) for peak detection.

Samples (25 μL of standard mixtures or natural extracts) were injected into the ion-exchange chromatography (IC) system using a mobile phase consisting of (A) Milli-Q water, (B) 1 M sodium hydroxide (NaOH), and (C) 1 M sodium acetate (NaOAc) at a flow rate of 5 mL/min. The chromatographic method was optimized for selective HIS separation and collection (Figure 2, SF2). An isocratic elution (60% A, 40% B) was applied for the first 33 min to achieve effective separation of HIS from other AAs, followed by a gradient elution (40% A, 10% B, 50% C) from 33 to 57 min to elute the remaining AAs. The system was re-equilibrated with 60% A and 40% B for 20 min before the next injection to ensure consistent performance.

**Figure 2:**
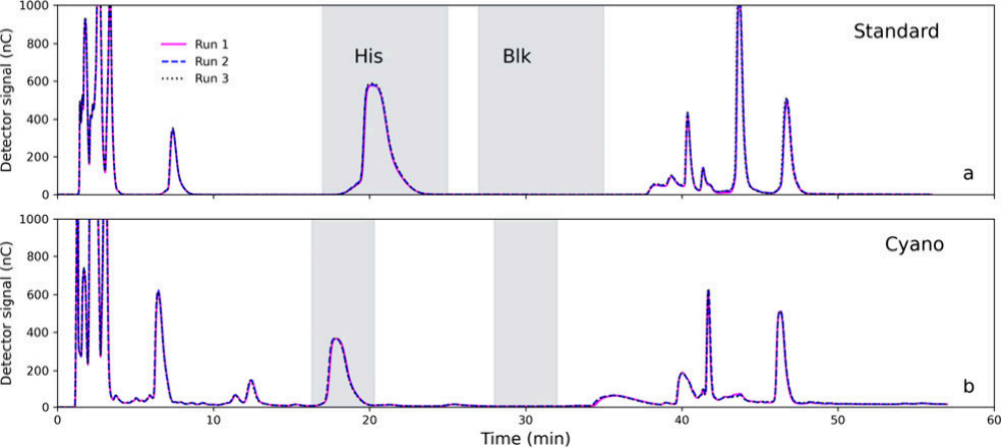
Selected ion-exchange chromatography (IC) chromatograms. (a) Standard mixture. (b) Cyanobacteria. Replicate injections were overlaid. The shaded areas denote the fraction collection windows of HIS and blank.

HIS was collected into precombusted glass vials, and a procedural blank with an equivalent collection window was included to account for potential isotopic interferences from potential coeluting nitrogenous compounds that could be oxidized to NO_3_^–^ or NO_2_^–^ during subsequent chemical processing (Figure 2). To assess the reproducibility of the analytical procedure, HIS from each standard mixture was analyzed in triplicate, while HIS from natural samples was analyzed in at least duplicate. HIS concentrations in natural samples were quantified by calibration against a series of standard mixtures (Figure SF3), which were used for calculating the oxidation yields in the following sections. Collected fractions were split into two aliquots (20 – 60 nmol of HIS in each aliquot) for oxidation (UV+POR or NaClO) (Figure 1).

### UV+POR Oxidation

For δ^15^N_HIS‑Total_ measurement, HIS was fully oxidized to NO_3_^–^ using UV-persulfate oxidation (POR) modified after Foreman et al.^36^ The persulfate reagent (0.185 M) was prepared by dissolving 1.25 g of recrystallized potassium persulfate (K_2_S_2_O_8_) (Fisher Chemical) and 0.4 g of NaOH (semiconductor grade, Honeywell, NC, USA) in 25 mL of Milli-Q water. Oxidation was performed in precombusted quartz vials (PurQ, Momentive Technologies) containing 20–80 nmol of HIS/urocanic acid standards in about 10 mL of Milli-Q water or 10 mL of IC-collected fractions, with 175 μL of POR reagent added. Samples were sealed with borosilicate stoppers and irradiated for 3 h in a UV oxidation chamber (7900, Ace Glass) equipped with a 1200-W mercury lamp (7825–40, Ace Glass), allowing the complete conversion of organic N to NO_3_^–^. Reaction blanks (Milli-Q water) were processed identically. The oxidation yields were verified by quantifying the NO_3_^–^ produced using a nutrient discrete analyzer (AQ300; Seal Analytical; US EPA Method 126-A). Oxidized samples were stored at 4 °C before isotope analysis.

### NaClO Oxidation

The α-N and a small portion of the side chain-N of HIS (as determined below) were converted to NH_3_ using sodium hypochlorite (NaClO) via Strecker degradation,^37^ which is then quantitatively converted to NO_2_^–^ under alkaline conditions (pH > 12), following established procedures.^38^ The procedures are detailed in the Supporting Information (SI Text 1).^30,38^ The concentration of the produced NO_2_^–^ was determined using a nutrient discrete analyzer (AQ300; Seal Analytical; US EPA Method 354.1).

### Conversion to N_2_O and δ^15^N Analysis

Reduction of NO_3_^–^ or NO_2_^–^ to N_2_O and the subsequent δ^15^N analysis was performed at University of Massachusetts Dartmouth (UMD). About 40 nmol of NO_3_^–^ obtained from UV+POR oxidation was reduced to N_2_O in serum vials by chemical conversion using Ti (III) following the procedures described in Altabet et al.^39^ Around 10 – 12 nmol of NO_2_^–^ in samples oxidized by NaClO were converted to N_2_O by sodium azide (NaN_3_) in serum vials following previously established procedures.^30,38,40,41^ δ^15^N analysis of N_2_O was conducted using a purge-and-trap continuous-flow isotope ratio mass spectrometer (PT/CF/IRMS; Isoprime Ltd.) following the established procedures.^39^ The analytical precision of δ^15^N–N_2_O measurement is ± 0.06‰. The details of the reference standards and calibration procedures are included in the SI Text S1.

### Data Correction and Calibration

Raw δ^15^N–N_2_O values were first corrected for blank contributions from the Ti (III) or NaN_3_ conversion and then calibrated against the isotopic reference standards following the protocol from Altabet et al.,^39^ outlined in the SI. To generate a calibration curve for δ^15^N_UV+POR_ values, values from pure HIS standards and IC-collected HIS standards were plotted against their independently measured δ^15^N values (δ^15^N_HIS‑Total_) from EA-IRMS:

1 $$\delta^{15}N_{\mathrm{UV}+\mathrm{POR}}=m\delta^{15}N_{\mathrm{HIS}‐\mathrm{Total}}+c$$

where *m* and *c* are the slope and *y*-intercept of the linear regression. The δ^15^N_UV+POR_ values of natural samples were then calibrated using eq 1 to yield δ^15^N_HIS‑Total_ values.

To estimate the δ^15^N values of α-N (δ^15^N_HIS‑α_) and side chain-N (δ^15^N_HIS‑s_, representing the average δ^15^N value of two imidazole N atoms), the following equations were applied:

2 $$\delta^{15}N_{\mathrm{ClO}}=r\left(\delta^{15}N_{\mathrm{His}‐\alpha }+\varepsilon_{\alpha }\right)+\left(1-r\right)\left(\delta^{15}N_{\mathrm{His}‐s}+\varepsilon_{s}\right)$$

3 $$\delta^{15}N_{\mathrm{HIS}‐\mathrm{Total}}=\frac{1}{3}\delta^{15}N_{\mathrm{His}‐\alpha }+\frac{2}{3}\delta^{15}N_{\mathrm{His}‐s}$$

where ε_α_ and ε_s_ represent fractionation factors for NaClO oxidation at the α-N and side chain-N sites, respectively. The fraction *r* represents the proportion of NO_2_^–^ derived from α-N oxidation by NaClO reaction. More details are included in the section “Estimation of δ^15^N_HIS‑α_ and δ^15^N_HIS‑s_ values” below.

## Results and Discussions

### Separation and Collection of HIS by IC

Baseline separation of HIS from other AAs in both standard mixtures and copepod samples was achieved using isocratic elution with NaOH and Milli-Q water during the first 33 min. The method demonstrated excellent retention time reproducibility, as evidenced by the overlaid chromatograms of replicate injections (Figure 2, SF2). The IC method was optimized for HIS separation and collection with a total runtime of less than 80 min. Key parameters such as injection volume, standard and sample concentrations, and the collection window width were carefully adjusted to ensure sufficient N mass was collected for subsequent UV + POR oxidation and δ^15^N analysis. Aliquots of each HIS fraction, along with IC procedural blanks, were directly subjected to UV + POR or NaClO oxidation without additional processing.

### UV+POR Oxidation and δ^15^N_HIS‑Total_ Values

Accurate determination of δ^15^N_HIS‑Total_ values without isotopic fractionation requires complete and quantitative oxidation of HIS-N to NO_3_^–^. Persulfate oxidation (POR) is a commonly used method for converting organic N to NO_3_^–^, but low recoveries (∼40–60%) have been reported for certain N-heterocyclic compounds, such as benzotriazole and antipyrine.^42,43^ We adopted the recommendation of Bronk et al.^42^ to use persulfate as the oxidant in UV oxidation (UV+POR), as persulfate can be readily activated by UV radiation.^44^ The mole ratio of persulfate to N in our protocol was ∼ 216:1 which was sufficient to fully oxidize HIS.^42,43,45,46^

To evaluate extraneous N introduced during sample preparation and oxidation, we quantified UV reaction blanks and IC procedural blanks. To minimize the background N in the persulfate reagent, K_2_S_2_O_8_ was recrystallized three times.^47^ The UV procedural blank in Milli-Q water measured 1.5 ± 0.4 μM, comparable to previous studies using POR alone (<2 μM) and UV/H_2_O_2_ (∼1.2 μM).^42^ IC procedural blanks collected from standard mixture injections exhibited slightly higher N concentrations, averaging 2.3 ± 0.5 μM.

After blank subtraction, oxidation yields of HIS isotopic standards in Milli-Q water and IC-purified HIS fractions from standard mixtures approached 100% following 3 h of UV exposure (Figure SF4, Table ST2). These consistently high yields confirm that HIS is quantitatively converted to NO_3_^–^ in both simple (Milli-Q) and IC matrices (NaOH). Correcting δ^15^N measurements of HIS isotopic standards with UV procedural blanks and IC-purified HIS fractions with paired IC procedural blanks yielded δ^15^N_UV+POR_ values that closely matched δ^15^N_HIS‑Total_ values obtained via EA-IRMS (Figure 3, SF5). Linear regression between δ^15^N_UV+POR_ and δ^15^N_HIS‑Total_ values measured by EA-IRMS produced slopes not significantly different from 1 (HIS standards: 1.00 ± 0.01; IC HIS standard fractions: 0.96 ± 0.01), with small intercepts (HIS standards: −0.78 ± 0.12; IC HIS fractions: 0.65 ± 0.15) and strong linearity (R^2^ > 0.999; Figure 3, SF5). These results confirm that accurate δ^15^N_HIS‑Total_ values are obtained when appropriate blank corrections are applied. Furthermore, they demonstrate that UV+POR effectively converts HIS-N to NO_3_^–^ with minimal fractionation, whether or not HIS is first separated via IC.

**Figure 3:**
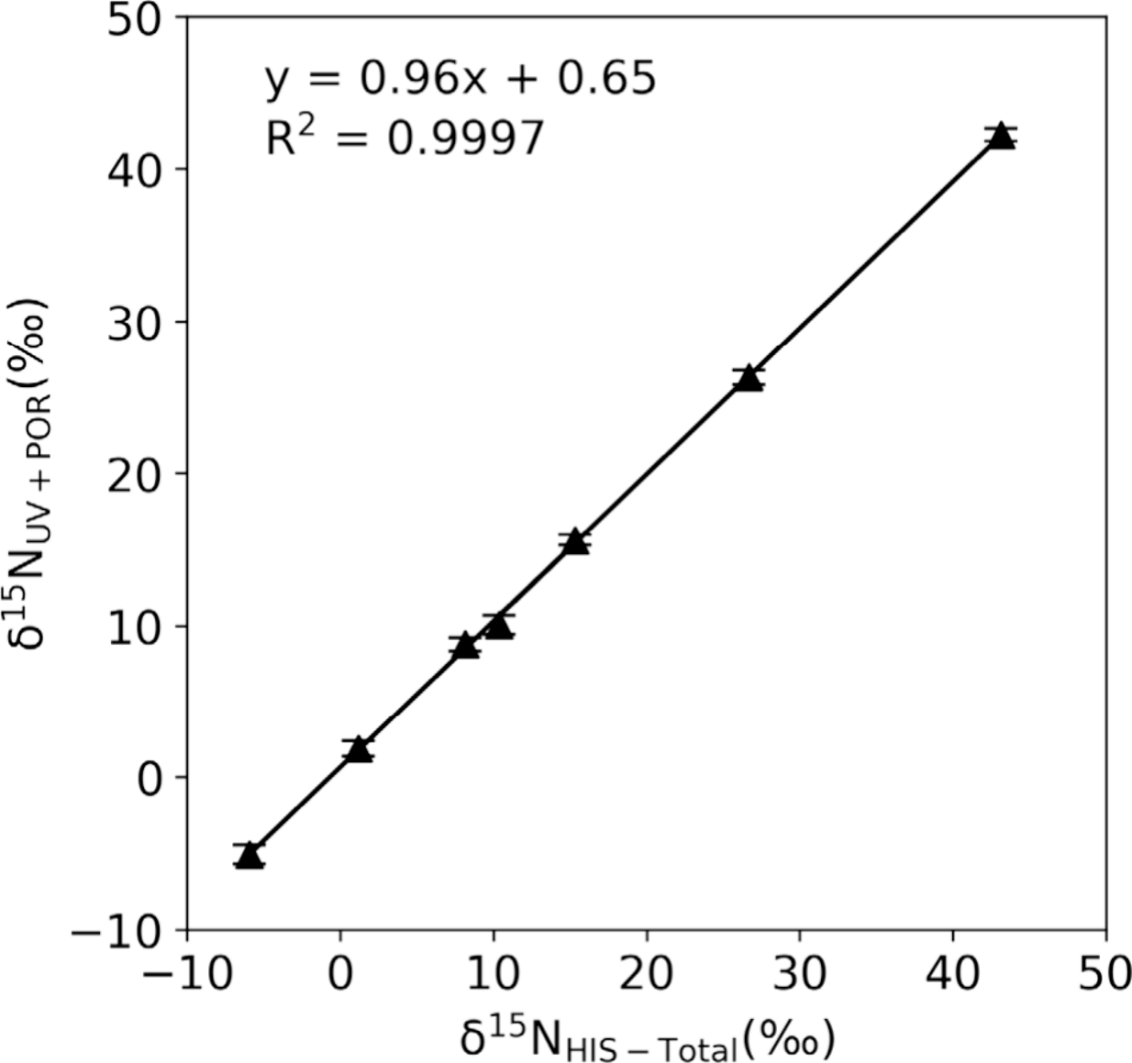
Comparison of δ^15^N values of HIS measured by two methods. δ^15^N_UV+POR_ values represent the values obtained from UV-persulfate oxidation of IC-collected HIS fractions, while δ^15^N_HIS‑Total_ values were determined independently by EA-IRMS (Table 1). The strong linear correlation (slope ≈ 1, small *y*-intercepts) demonstrates excellent agreement between the two methods for determining the molecular average δ^15^N values of HIS.

For the natural samples, propagated error of δ^15^N_HIS‑Total_ values from instrument precision, blank correction, and calibration with eq 1 was generally below 0.2‰ (SI Text S2), which is lower than the observed standard deviation of δ^15^N_HIS‑Total_ measurements from replicate IC injections (±0.1‰ – 1.3‰). Consequently, the standard deviation of replicates is reported as the uncertainty for δ^15^N_HIS‑Total_ values of natural samples in Table 2.

**Table 2:** δ^15^N_HIS‑α_ and δ^15^N_HIS‑s_ Values of Natural Samples Calculated from the Values of δ^15^N_UV+POR_ (Calibrated to δ^15^N_HIS‑Total_) and δ^15^N_ClO_ of IC-Collected HIS Fractions^a^

Sample	δ^15^N_HIS‑Total_ (‰)	δ^15^N_ClO_ (‰)	δ^15^N_HIS‑s_ (‰)	δ^15^N_HIS‑α_ (‰)	δ^15^N_Phe_ (‰)^a^	δ^15^N_Glu_ (‰)^a^
Cyanobacteria	5.2 ± 0.8	6.9 ± 0.2	4.3 ± 1.4	7.1 ± 1.6	7.9 ± 0.4	9.0 ± 0.4
Copepod	2.5 ± 0.1	14.6 ± 0.5	–5.4 ± 0.5	18.4 ± 0.5	2.6 ± 0.2	14.5 ± 0.1
Euphausiid	2.7 ± 0.1	15.3 ± 1.0	–5.6 ± 0.8	19.3 ± 0.8	2.0 ± 0.6	18.6 ± 0.7
Fish	4.9 ± 1.3	11.5 ± 0.3	0.7 ± 2.2	13.3 ± 2.5	2.6 ± 0.4	17.8 ± 0.3
POM	3.6 ± 0.4	3.3 ± 0.3	3.9 ± 0.8	2.8 ± 0.9	3.4 ± 0.5	9.9 ± 0.5

a The δ^15^N_Phe_ and δ^15^N_Glu_ values were analyzed with the method described by Zhang et al.^30^.

The precision achieved in this study is comparable to δ^15^N_HIS‑Total_ uncertainties obtained from GC/C/IRMS for biological samples (±0.4‰ to ± 2.6‰)^13,15,16^ and within the uncertainty range of δ^15^N values for other AAs analyzed by CSIA-AA methods (∼ ± 0.13‰ to ± 1‰).^23,48^ These findings suggest our method is a reliable approach for determining δ^15^N_HIS‑Total_ values with high accuracy and precision.

### Estimation of δ^15^N_HIS‑α_ and δ^15^N_HIS‑s_ Values

To estimate the δ^15^N_HIS‑α_ and δ^15^N_HIS‑s_ values, ε_α_, ε_s_, and r in eq 2 are needed. ε_α_ was assumed to be similar to the fractionation factor for NaClO oxidation of phenylalanine (Phe), which is 0.6‰.^30^ Phe was chosen as an analog for estimating the ε_α_ of HIS due to its similar NaClO oxidation yield (∼70%;^30^ Table ST3) and aromatic side chain structure. ε_s_ was obtained by the difference between the δ^15^N_HIS‑Total_ value obtained from UV+POR oxidation and δ^15^N_ClO_ value of urocanic acid, a structural analog of HIS’s side chain. ε_s_ was determined as −1.3‰ given δ^15^N_HIS‑Total_ = 3.8 ± 0.8‰ and δ^15^N_ClO_ = 2.5 ± 0.9‰.

r represents the proportion of NO_2_^–^ derived from α-N oxidation by NaClO, while (1 – r) corresponds to the fraction originating from side chain-N. It was previously reported that a small fraction of side chain N from poly-N AAs such as HIS, arginine, tryptophan, and asparagine can be oxidized to NO_2_^–^ by NaClO.^38^ In our study, we also observed a small amount of NO_2_^–^ produced during the NaClO oxidation of urocanic acid (Table ST3). r was estimated by plotting δ^15^N_ClO_ against δ^15^N_HIS‑Total_ for the α-series HIS isotopic standards:

4 $$\delta^{15}N_{\mathrm{ClO}}=m_{\alpha ,\mathrm{ClO}}\delta^{15}N_{\mathrm{HIS}‐\mathrm{Total}}+c_{\alpha ,\mathrm{ClO}}$$

where *m*_α,ClO_ and *c*_α,ClO_ are the slope and *y*-intercept, respectively. Since the δ^15^N_HIS‑s_ value remains constant (δ^15^N_HIS‑s0_) within the α-series, the only unknowns in eq 2 are r and δ^15^N_HIS‑α_. By substituting eq 3 into eq 4:

5 $$\delta^{15}N_{\mathrm{ClO}}=\frac{m_{a,\mathrm{ClO}}}{3}\delta^{15}N_{\mathrm{His}‐\alpha }+\frac{2m_{a,\mathrm{ClO}}}{3}\delta^{15}N_{\mathrm{His}‐s0}+c_{\alpha ,\mathrm{ClO}}$$

By comparing eq 5 with eq 2,

6 $$r=\frac{m_{a,\mathrm{ClO}}}{3}$$

*r* was determined to be 0.83 for IC-purified HIS fractions of standard mixtures, based on *m*_α, ClO_ (2.49) in Figure 4. Substituting *r* = 0.83, ε_α_ = +0.6‰, and ε_s_ = −1.3‰ into eq 2, eq 7 is derived as

7 $$\delta^{15}N_{\mathrm{ClO}}=0.83\left(\delta^{15}N_{\mathrm{His}‐\alpha }+0.6\right)+0.17\left(\delta^{15}N_{\mathrm{His}‐s}-1.3\right)$$

**Figure 4:**
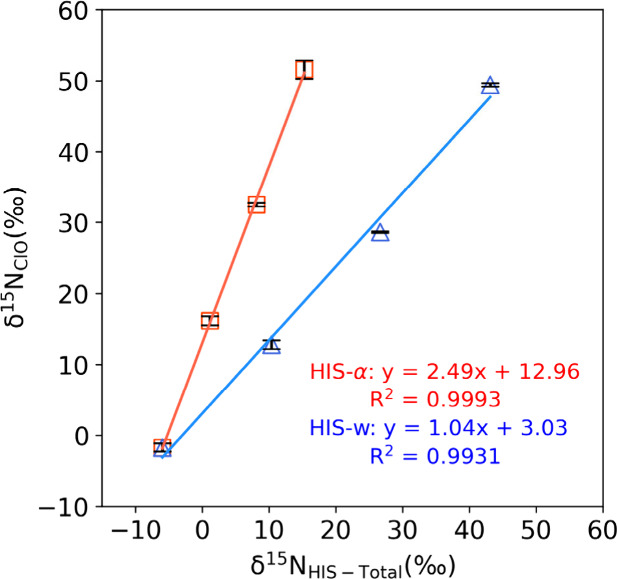
δ^15^N_ClO_ values (measured from NaClO oxidation) of HIS fractions collected from IC injections of standard mixtures are plotted against their δ^15^N_HIS‑Total_ values determined by EA-IRMS (Table 1). δ^15^N_ClO_ values reflect a weighted average of the δ^15^N values from both the α-N and side chain-N positions. The slope of the HIS-α series was used to calculate the proportion of NO_2_^–^ produced from α-N, according to eqs 4 and 6 in the main text. The *y*-intercept of the HIS-α series can be expressed as *c*_α_,_ClO_ = (1 – 3*r*) δ^15^N_His‑s0_ + *r* ε_α_ + (1 – *r*) ε_s_, where *r* is the fraction of NO_2_^–^ derived from α-N, and ε_α_ and ε_s_ are the fractionation factors for NaClO oxidation at the α- and side chain-N positions, respectively. If NaClO oxidation were specific only to the α-N, the slope of the HIS-α series (in which only δ^15^N_HIS‑α_ increases) would be 3. Observed slopes < 3 suggest a minor but measurable contribution from side chain-N to the δ^15^N_ClO_ values.

The values of δ^15^N_HIS‑s_ and δ^15^N_HIS‑α_ could thus be estimated by eqs 3 and 7. The combined contribution of ε_α_ (0.6‰) and ε_s_ (−1.3‰) to the estimation of δ^15^N_HIS‑s_ and δ^15^N_HIS‑α_ values would be +0.28‰ only, such that the δ^15^N_HIS‑s_ and δ^15^N_HIS‑α_ values would be mostly driven by δ^15^N_HIS‑Total_ and δ^15^N_ClO_ values.

(1 – r) is 0.17, suggesting that ∼17% of the total oxidized NO_2_^–^ product originated from the side chain-N of IC-collected HIS fractions. Considering an oxidation yield of ∼70–80% from HIS molecules to NO_2_^–^ in all HIS standards and natural samples (Table ST3), NO_2_^–^ product derived from the side chain-N of IC-collected HIS was ∼12–14% per HIS molecule, which is significantly higher than the amount of NO_2_^–^ yielded per urocanic acid molecule in NaOH (∼3%; Table ST3). The enhanced oxidation of HIS side chain-N, compared to urocanic acid, may be attributed to the presence of α-N or the formation of a Strecker aldehyde with an imidazole side chain during HIS degradation.^49^ This likely increases the susceptibility of the side chain-N to oxidation, leading to a higher fraction of side chain-derived NO_2_^–^ in HIS relative to urocanic acid, which does not form Strecker aldehyde during NaClO oxidation.

The estimated δ^15^N_HIS‑α_ and δ^15^N_HIS‑s_ values for HIS isotopic standards are shown in Table 1. The α-series exhibited δ^15^N_HIS‑s_ values (∼ −8 to −9‰) that were indistinguishable from HIS-0, confirming that only the α-N was enriched in ^15^N. In contrast, the w-series showed increases in both δ^15^N_HIS‑α_ and δ^15^N_HIS‑s_ values, consistent with the isotopic labeling of all N atoms in this series.

The precision of δ^15^N_ClO_ measurements in HIS fractions from replicate IC injections of standard mixtures and natural samples was generally better than ± 1.3‰, with most samples showing uncertainties of <±0.7‰ (Tables 1 and 2). Errors in δ^15^N_HIS‑α_ and δ^15^N_HIS‑s_ values, propagated from uncertainties in δ^15^N_HIS‑Total_, δ^15^N_ClO_ values, and r with the procedures listed in SI Text S2, ranged from ±0.4–1.0‰ for standard mixtures and ±0.5–1.6‰ for most natural samples. The overall precision of δ^15^N_HIS‑α_ and δ^15^N_HIS‑s_ measurements obtained in this study is comparable to reported uncertainties in δ^15^N_HIS‑Total_ values for biological samples analyzed using GC/C/IRMS.^13,15,16^ These results support the reliability of our analytical scheme for distinguishing α- and side chain-N isotopic compositions in HIS.

### Molecular Average and Intramolecular δ^15^N_HIS_ Values of Natural Samples

The δ^15^N_HIS‑Total_ values of natural samples ranged from 2.5 – 5.2‰ (Table 2), consistent with previously reported δ^15^N_HIS‑Total_ values in biological samples analyzed using GC/C/IRMS (−2.4 – 6.8‰).^13–16^ To provide further context, δ^15^N values of the source AA phenylalanine (Phe) and the trophic AA glutamic acid (Glu) were also measured in the same samples using the IC × PT-CF-IRMS method.^30^ Across all samples, δ^15^N_HIS‑Total_ values were lower than δ^15^N_Glu_ values by ∼4–16‰ but within ±3‰ of δ^15^N_Phe_ values. This pattern is consistent with previous observations in marine consumers, where δ^15^N_HIS‑Total_ values closely track δ^15^N_Phe_ values (<±5‰) but remain significantly lower than δ^15^N_Glu_ values (>10‰).^13,15,16^ This trend reflects the extensive transamination that enriches Glu in ^15^N compared to source AAs, particularly with increasing trophic levels.^6,33,50^

Intramolecular δ^15^N heterogeneity within HIS molecules was observed in the HIS-0 standard and all biological samples, except for POM (Table 2, Figure SF7). The Δδ^15^N_α‑s_ (δ^15^N_HIS‑α_ – δ^15^N_HIS‑s_) of HIS-0 was approximately +8‰. This contrasts with the average Δδ^15^N_α‑s_ of −9‰ reported by Sacks and Brenna^26^ for commercial HIS standards from four different vendors (Acros, Belgium; Avocado Organics, UK; J.T. Baker, USA; Sigma, USA), which were analyzed by enzymatic cleavage of α-N followed by GC/C/IRMS. Commercial l-histidine is typically produced via microbial fermentation,^51^ meaning it retains a biological isotopic signature. The opposing Δδ^15^N_α‑s_ sign in our HIS-0 standard may be caused by the differences in manufacturing processes of HIS adopted by different vendors. However, the consistent observation of positive Δδ^15^N_α‑s_ across biological samples in this study suggests a systematic isotopic fractionation pattern related to N metabolism.

One of HIS’ side chain-N is from ATP’s adenine moiety (Figure SF1a). Previous studies show that N in heterocyclic rings (purines/pyrimidines) is generally ^15^N-depleted compared to the bulk protein N pool.^52,53^ For example, Strable et al.^52^ found that in mammalian cerebellar DNA, the purine bases had δ^15^N values of about – 7 to −10‰, whereas bulk DNA was −4.5‰ and whole tissue was +4.1‰, suggesting purine N was more depleted in ^15^N than the amino-acid-derived N. Likewise, Broek et al.^54^ reported that heterocyclic-N structures in marine dissolved organic N are distinctly ^15^N-depleted relative to amide-N and amine-N. Together, these studies suggest that the side chain-N in HIS originating from an ATP-derived heterocycle (adenine) should carry a lower δ^15^N signal than the α-N (from Glu) equilibrates with the cellular amino-N pool, which is often enriched in ^15^N at higher trophic levels. This isotope patterns explain our finding of a positive Δδ^15^N_α‑s_ (+2.8‰) in our cyanobacteria sample. We also note that HIS biosynthesis differs between prokaryotes and eukaryotes, which could affect the isotopic patterns in HIS. In prokaryotes like cyanobacteria, the imidazole ring of HIS is catalyzed by a two-step enzyme, whereas many eukaryotes complete this step using a single bifunctional enzyme.^9,55^ If such structural differences alter ^15^N fractionation, then cyanobacteria (prokaryotic) versus algal/plant (eukaryotic) might have different intramolecular δ^15^N_HIS_ values, which warrants further investigation.

POM in the surface ocean primarily consists of phytoplankton, heterotrophic bacteria, and detrital organic matter. The small Δδ^15^N_α‑s_ in POM (−1.1‰) may result from an integration of biosynthetic signals from different microbial sources and isotopic fractionation during environmental degradation. Compared to cyanobacteria and POM, the three consumer samples exhibited markedly higher Δδ^15^N_α‑s_ values (fish: +12.6‰, copepods and euphausiids: +23 to +25‰; Table 2, Figure SF7), with δ^15^N_HIS‑α_ values elevated relative to δ^15^N_Phe_ values and approaching δ^15^N_Glu_ values. The substantial ^15^N enrichment of α-N in consumers is surprising given the limited exchange between HIS α-N and the metabolic N pool. Since higher trophic organisms cannot synthesize HIS de novo, it is unlikely that metabolic N directly contributes to HIS formation or the ^15^N enrichment of α-N. Therefore, it is unlikely that δ^15^N_HIS‑α_ values are tightly coupled with the values of δ^15^N_Glu_ or δ^15^N of the metabolic N pool.

Instead, we hypothesize that δ^15^N_HIS‑α_ values in consumers are influenced by dietary inputs and the extent of HIS catabolism. The N isotopic fractionation associated with histidase-mediated deamination of HIS has not been directly studied, but similar ammonia-lyases that cleave the α-N of Phe preferentially remove ^14^N, leading to ^15^N enrichment in the residual Phe.^56^ If HIS deamination follows this pattern, it would increase δ^15^N_HIS‑α_ values while having minimal effect on δ^15^N_HIS‑s_ values, as the side chain-N does not participate in deamination. HIS catabolic rates are linked to physiological and environmental factors such as starvation and N limitation, both of which can decrease HIS concentrations in diatom and fish.^57,58^ Additionally, N isotopic turnover and HIS catabolic rates are typically higher in organs than in muscle tissues.^57,59^ Thus, interspecies differences in Δδ^15^N_α‑s_ among the consumers may reflect variations in diet quality, growth stage, physiological status, protein turnover rates, and tissue type.^60–63^ Notably, the strong ^15^N enrichment in consumer α-N of HIS has not been previously identified because the δ^15^N_HIS‑α_ signal is diluted by lower δ^15^N_HIS‑s_ values, resulting in δ^15^N_HIS‑Total_ values that remain comparable to δ^15^N_Phe_ values.

## Summary

This study validates an analytical framework for measuring δ^15^N values of poly-N amino acids at both compound-specific and intramolecular levels, using histidine (HIS) as a model. We demonstrated that UV+POR oxidation efficiently converts HIS to NO_3_^–^ in various matrices, including Milli-Q water and NaOH. When combined with NaClO oxidation, which preferentially targets α-N, our approach will enable intramolecular δ^15^N analysis of HIS. This approach can be extended to other poly-N AAs such as arginine, glutamine, asparagine, lysine, and tryptophan. With appropriate isotopic standards, the δ^15^N values of α-N and side chain-N in these amino acids can be determined as well.

Beyond the utility of δ^15^N-AAs in food web studies, where differential trophic ^15^N enrichment among AAs helps infer trophic relationships, intramolecular δ^15^N analysis of poly-N AAs provides valuable insights into N metabolism and biochemical pathways. Potential applications include tracing nucleobase synthesis from the amide-N of glutamine,^53^ investigating the role of arginine’s guanidino-N in the urea cycle,^64^ and examining the formation of indole during tryptophan biosynthesis—a signaling molecule that regulates many physiological functions.^65,66^

## Supplementary Material



## Notes

Additional details on biosynthetic pathways, methods, calculations, and results (PDF)

## #

Woods Hole Oceanographic Institution, Woods Hole, Massachusetts 02543, United States

## References

[1] Chikaraishi Y., Steffan S. A., Ogawa N. O., Ishikawa N. F., Sasaki Y., Tsuchiya M., Ohkouchi N. (2014). High-resolution food webs based on
nitrogen isotopic composition of amino acids. Ecol. Evol..

[2] McCarthy M. D., Lehman J., Kudela R. (2013). Compound-specific amino acid δ15N
patterns in marine algae: Tracer potential for cyanobacterial vs.
eukaryotic organic nitrogen sources in the ocean. Geochim. Cosmochim. Acta.

[3] Mompeán C., Bode A., Gier E., McCarthy M. D. (2016). Bulk vs.
amino acid
stable N isotope estimations of metabolic status and contributions
of nitrogen fixation to size-fractionated zooplankton biomass in the
subtropical N Atlantic. Deep Sea Res., Part
I.

[4] McMahon K. W., McCarthy M. D. (2016). Embracing variability
in amino acid δ15N fractionation:
mechanisms, implications, and applications for trophic ecology. Ecosphere.

[5] Vokhshoori N. L., McCarthy M. D., Close H. G., Demopoulos A. W. J., Prouty N. G. (2021). New geochemical tools for investigating resource and
energy functions at deep-sea cold seeps using amino acid δ15N
in chemosymbiotic mussels (Bathymodiolus childressi). Geobiology.

[6] Ohkouchi, N. ; Takano, Y. Organic Nitrogen: Sources, Fates, and Chemistry. In Treatise on Geochemistry (Second ed.), Holland, H. D. , Turekian, K. K. , Eds.; Elsevier, 2014; pp 251–289.

[7] Ohkouchi N., Chikaraishi Y., Close H. G., Fry B., Larsen T., Madigan D. J., McCarthy M. D., McMahon K. W., Nagata T., Naito Y. I. (2017). Advances in the application of amino acid nitrogen
isotopic analysis in ecological and biogeochemical studies. Org. Geochem..

[8] Alifano P., Fani R., Liò P., Lazcano A., Bazzicalupo M., Carlomagno M. S., Bruni C. B. (1996). Histidine biosynthetic pathway and
genes: structure, regulation, and evolution. Microbiol.l Rev..

[9] Stepansky A., Leustek T. (2006). Histidine biosynthesis in plants. Amino Acids.

[10] O’Connell T. C. (2017). ’Trophic’
and ’source’ amino acids in trophic estimation: a likely
metabolic explanation. Oecologia.

[11] Aqvist S. E. G. (1951). Amino acid interrelationships during growth, studied
with N15-labeled glycine in regenerating rat liver. Acta Chem. Scand..

[12] Metges C. C., Petzke K.-J., Hennig U. (1996). Gas Chromatography/Combustion/Isotope
Ratio Mass Spectrometric Comparison of N -Acetyl- and N-Pivaloyl Amino
Acid Esters to Measure 15N Isotopic Abundances in Physiological Samples:
A Pilot Study on Amino Acid Synthesis in the Upper Gastro-intestinal
Tract of Minipigs. J. Mass Spectrom..

[13] Lorrain A., Graham B., Ménard F., Popp B., Bouillon S., Van Breugel P., Cherel Y. (2009). Nitrogen and carbon isotope values
of individual amino acids: a tool to study foraging ecology of penguins
in the Southern Ocean. Mar. Ecol.: Prog. Ser..

[14] Petzke K. J., Boeing H., Klaus S., Metges C. C. (2005). Carbon and Nitrogen
Stable Isotopic Composition of Hair Protein and Amino Acids Can Be
Used as Biomarkers for Animal-Derived Dietary Protein Intake in Humans12. J. Nutr..

[15] Popp B. N., Graham B. S., Olson R. J., Hannides C. C. S., Lott M. J., López-Ibarra G. A., Galván-Magaña F., Fry B. (2007). Insight into the Trophic
Ecology of Yellowfin Tuna, Thunnus albacares,
from Compound-Specific Nitrogen Isotope Analysis of Proteinaceous
Amino Acids. Terrestrial Ecology.

[16] Schmidt K., Atkinson A., Petzke K.-J., Voss M., Pond D. W. (2006). Protozoans
as a food source for Antarctic krill, Euphausia superba: Complementary
insights from stomach content, fatty acids, and stable isotopes. Limnol. Oceanogr..

[17] Brenner, M. ; Ames, B. N. The Histidine Operon and Its Regulation. In Metabolic Regulation (Third ed.), Vogel, H. J. , Ed.; Academic Press, 1971; pp 349–387.

[18] Yamaguchi Y. T., Chikaraishi Y., Takano Y., Ogawa N. O., Imachi H., Yokoyama Y., Ohkouchi N. (2017). Fractionation of nitrogen isotopes
during amino acid metabolism in heterotrophic and chemolithoautotrophic
microbes across Eukarya, Bacteria, and Archaea: Effects of nitrogen
sources and metabolic pathways. Org. Geochem..

[19] Bender R. A. (2012). Regulation
of the Histidine Utilization (Hut) System in Bacteria. Microbiol. Mol. Biol. Rev..

[20] Polkinghorne M. A., Hynes M. J. (1982). L-histidine utilization
in Aspergillus nidulans. J. Bacteriol..

[21] Hofmann D., Gehre M., Jung K. (2003). Sample preparation techniques for
the determination of natural 15N/14N variations in amino acids by
gas chromatography-combustion-isotope ratio mass spectrometry (GC-C-IRMS). Isot. Env. Health Stud..

[22] Ishikawa N. F., Itahashi Y., Blattmann T. M., Takano Y., Ogawa N. O., Yamane M., Yokoyama Y., Nagata T., Yoneda M., Haghipour N. (2018). Improved Method for Isolation and Purification
of Underivatized Amino Acids for Radiocarbon Analysis. Anal. Chem..

[23] Ishikawa N. F., Ogawa N. O., Sun Y., Chikaraishi Y., Takano Y., Ohkouchi N. (2022). Integrative assessment
of amino acid
nitrogen isotopic composition in biological tissue samples determined
by GC/C/IRMS, LC × EA/IRMS, and LC × GC/C/IRMS. Limnol. Oceanogr. Methods.

[24] Walsh R. G., He S., Yarnes C. T. (2014). Compound-specific
δ13C and δ15N analysis
of amino acids: a rapid, chloroformate-based method for ecological
studies. Rapid Commun. Mass Spectrom..

[25] Silverman S. N., Phillips A. A., Weiss G. M., Wilkes E. B., Eiler J. M., Sessions A. L. (2022). Practical
considerations for amino acid isotope analysis. Org. Geochem..

[26] Sacks G. L., Brenna J. T. (2005). 15N/14N Position-Specific Isotopic Analyses of Polynitrogenous
Amino Acids. Anal. Chem..

[27] Broek T. A. B., Walker B. D., Andreasen D. H., McCarthy M. D. (2013). High-precision measurement
of phenylalanine δ15N values for environmental samples: A new
approach coupling high-pressure liquid chromatography purification
and elemental analyzer isotope ratio mass spectrometry. Rapid Commun. Mass Spectrom..

[28] Sun Y., Ogawa N. O., Ishikawa N. F., Blattmann T. M., Takano Y., Ohkouchi N. (2023). Application of a porous
graphitic
carbon column to carbon and nitrogen isotope analysis of underivatized
individual amino acids using high-performance liquid chromatography
coupled with elemental analyzer/isotope ratio mass spectrometry. Rapid Commun. Mass Spectrom..

[29] Broek T. A. B., McCarthy M. D. (2014). A new approach to
δ15N compound-specific amino
acid trophic position measurements: preparative high pressure liquid
chromatography technique for purifying underivatized amino acids for
stable isotope analysis. Limnol. Oceanogr. Methods.

[30] Zhang L., Lee W.-m., Kreider-Mueller A., Kuhnel E., Baca J., Ji C., Altabet M. (2021). High-precision measurement of phenylalanine and glutamic
acid δ15N by coupling ion-exchange chromatography and purge-and-trap
continuous-flow isotope ratio mass spectrometry. Rapid Commun. Mass Spectrom..

[31] Sánchez-Velasco L., García-De
León F. J., Ruvalcada-Aroche E. D., Beier E., Godínez V. M., Jiménez-Rosenberg S. P. A., Sánchez-Pérez E. D., Contreras-Catala F., Mnich A., Verma N. (2022). Vertical
distribution
of zooplankton groups, with an emphasis on fish larvae, in the oxygen
minimum zone off southern México (December 2020). J. Mar. Sys..

[32] Lee C. W. M., Altabet M., Mnich A., Zhang L. (2025). Using δ15N of
Amino Acids and Nitrate to Investigate Particle Production and Transformation
in the Ocean: A Case Study From the Eastern Tropical North Pacific
Oxygen Deficient Zone. Global Biogeochem. Cycles.

[33] Chikaraishi Y., Ogawa N. O., Kashiyama Y., Takano Y., Suga H., Tomitani A., Miyashita H., Kitazato H., Ohkouchi N. (2009). Determination
of aquatic food-web structure based on compound-specific nitrogen
isotopic composition of amino acids. Limnol.
Oceanogr. Methods.

[34] McCarthy M. D., Benner R., Lee C., Fogel M. L. (2007). Amino acid nitrogen
isotopic fractionation patterns as indicators of heterotrophy in plankton,
particulate, and dissolved organic matter. Geochim.
Cosmochim. Acta.

[35] Takano Y., Kashiyama Y., Ogawa N. O., Chikaraishi Y., Ohkouchi N. (2010). Isolation and desalting
with cation-exchange chromatography
for compound-specific nitrogen isotope analysis of amino acids: application
to biogeochemical samples. Rapid Commun. Mass
Spectrom..

[36] Foreman R. K., Björkman K. M., Carlson C. A., Opalk K., Karl D. M. (2019). Improved
ultraviolet photo-oxidation system yields estimates for deep-sea dissolved
organic nitrogen and phosphorus. Limnol. Oceanogr.
Methods.

[37] Schonberg A., Moubacher R. (1952). The Strecker Degradation of α-Amino Acids. Chem. Rev..

[38] Zhang L., Altabet M. A. (2008). Amino-group-specific natural abundance nitrogen isotope
ratio analysis in amino acids. Rapid Commun.
Mass Spectrom..

[39] Altabet M. A., Wassenaar L. I., Douence C., Roy R. (2019). A Ti­(III) reduction
method for one-step conversion of seawater and freshwater nitrate
into N2O for stable isotopic analysis of 15N/14N, 18O/16O and 17O/16O. Rapid Commun. Mass Spectrom..

[40] McIlvin M. R., Altabet M. A. (2005). Chemical Conversion
of Nitrate and Nitrite to Nitrous
Oxide for Nitrogen and Oxygen Isotopic Analysis in Freshwater and
Seawater. Anal. Chem..

[41] Zhang L., Altabet M. A., Wu T., Hadas O. (2007). Sensitive Measurement
of NH4+ 15N/14N (δ15NH4+) at Natural Abundance Levels in Fresh
and Saltwaters. Anal. Chem..

[42] Bronk D. A., Lomas M. W., Glibert P. M., Schukert K. J., Sanderson M. P. (2000). Total dissolved
nitrogen analysis: comparisons between the persulfate, UV and high
temperature oxidation methods. Mar. Chem..

[43] Nydahl F. (1978). On the peroxodisulphate
oxidation of total nitrogen in waters to nitrate. Water Res..

[44] Yang Q., Ma Y., Chen F., Yao F., Sun J., Wang S., Yi K., Hou L., Li X., Wang D. (2019). Recent advances in
photo-activated sulfate radical-advanced oxidation process (SR-AOP)
for refractory organic pollutants removal in water. Chem. Eng. J..

[45] Yang S., Wang P., Yang X., Shan L., Zhang W., Shao X., Niu R. (2010). Degradation efficiencies of azo dye
Acid Orange 7 by the interaction of heat, UV and anions with common
oxidants: Persulfate, peroxymonosulfate and hydrogen peroxide. J. Hazard. Mater..

[46] Knapp, A. N. ; Sigman, D. M. ; Lipschultz, F. N isotopic composition of dissolved organic nitrogen and nitrate at the Bermuda Atlantic Time-series Study site. Global Biogeochem. Cycles 2005, 19 (1) 10.1029/2004GB002320.

[47] Solórzano L., Sharp J. H. (1980). Determination of
total dissolved nitrogen in natural
waters. Limnol. Oceanogr..

[48] Yarnes C. T., Herszage J. (2017). The relative influence
of derivatization and normalization
procedures on the compound-specific stable isotope analysis of nitrogen
in amino acids. Rapid Commun. Mass Spectrom..

[49] Zamora R., Delgado R. M., Hidalgo F. J. (2011). Strecker
aldehydes and α-keto
acids, produced by carbonyl-amine reactions, contribute to the formation
of acrylamide. Food Chem..

[50] McClelland J. W., Montoya J. P. (2002). Trophic relationships
and the nitrogen isotopic composition
of amino acids in plankton. Ecology.

[51] Becker J., Wittmann C. (2012). Bio-based production of chemicals,
materials and fuels
- Corynebacterium glutamicum as versatile cell factory. Curr. Opin. Biotechnol..

[52] Strable M. S., Tschanz C. L., Varamini B., Chikaraishi Y., Ohkouchi N., Brenna J. T. (2011). Mammalian DNA δ15N
exhibits
40‰ intramolecular variation and is unresponsive to dietary
protein level. Rapid Commun. Mass Spectrom..

[53] Koga T., Takano Y., Ogawa N. O., Hollingsworth E. H., Oba Y., Ohkouchi N. (2025). Compound-Specific Carbon
and Nitrogen Isotopic Analyses
of Underivatized Pyrimidine and Purine Nucleobases. ACS Earth and Space Chem..

[54] Broek T. A. B., McCarthy M. D., Ianiri H. L., Vaughn J. S., Mason H. E., Knapp A. N. (2023). Dominant heterocyclic
composition of dissolved organic
nitrogen in the ocean: A new paradigm for cycling and persistence. Proc. Natl. Acad. Sci. U. S. A..

[55] Fani R., Brilli M., Fondi M., Lió P. (2007). The role of
gene fusions in the evolution of metabolic pathways: the histidine
biosynthesis case. BMC Evol. Biol..

[56] Hermes J. D., Weiss P. M., Cleland W. W. (1985). Use of
nitrogen-15 and deuterium
isotope effects to determine the chemical mechanism of phenylalanine
ammonia-lyase. Biochemistry.

[57] Abe H., Brill R. W., Hochachka P. W. (1986). Metabolism
of L-Histidine, Carnosine,
and Anserine in Skipjack Tuna. Physiol. Zool..

[58] Pan Y., Hu F., Yu C., Li C., Huang T., Hu H. (2020). Amino Acid
Catabolism During Nitrogen Limitation in Phaeodactylum tricornutum. Front. Plant Sci..

[59] Madigan D. J., Litvin S. Y., Popp B. N., Carlisle A. B., Farwell C. J., Block B. A. (2012). Tissue Turnover Rates and Isotopic Trophic Discrimination
Factors in the Endothermic Teleost, Pacific Bluefin Tuna (Thunnus
orientalis). PLoS One.

[60] Bradley C. J., Madigan D. J., Block B. A., Popp B. N. (2014). Amino Acid Isotope
Incorporation and Enrichment Factors in Pacific Bluefin Tuna, Thunnus
orientalis. PLoS One.

[61] Conceição L. E. C., van
der Meeren T., Verreth J. A. J., Evjen M. S., Houlihan D. F., Fyhn H. J. (1997). Amino acid metabolism and protein
turnover in larval turbot (Scophthalmus maximus) fed natural zooplankton
or Artemia. Mar. Biol..

[62] Kaushik S. J., Seiliez I. (2010). Protein and amino acid
nutrition and metabolism in
fish: current knowledge and future needs. Aquaculture
Research.

[63] Ventura M., Catalan J. (2010). Variability in amino
acid composition of alpine crustacean
zooplankton and its relationship with nitrogen-15 fractionation. J. Plankton Res..

[64] Kendall I. P., Evershed R. P. (2020). Determination of
Arginine δ15N Values in Plant
and Animal Proteins by Gas Chromatography-Combustion-Isotope Ratio
Mass Spectrometry. Anal. Chem..

[65] Fiore A., Murray P. J. (2021). Tryptophan and indole metabolism in immune regulation. Curr. Opin. Immunol..

[66] Palego L., Betti L., Rossi A., Giannaccini G. (2016). Tryptophan
Biochemistry: Structural, Nutritional, Metabolic, and Medical Aspects
in Humans. J. Amino Acids.

