# Supplementary material for: Compound-Specific and Intramolecular δ15N Analysis of a Poly-Nitrogenous Amino Acid: Histidine
Source: Anal Chem. 2025 Jul 24;97(30):16288–95. doi: 10.1021/acs.analchem.5c01711 (PMC12332842; doi:10.1021/acs.analchem.5c01711)
Supplement: Supplementary file 1 [file ac5c01711_si_001.pdf]

Supporting Information for

Compound-Specific and Intramolecular $\delta^{15}N$ analysis of a Poly-Nitrogenous Amino Acid: Histidine.

Charlotte Wing Man Lee[1†], Mark A. Altabet[2], Jesus Baca[1], Jason Barrera[1], and Lin Zhang[1*]

(1) Texas A&M University-Corpus Christi, Corpus Christi, TX, 78412, United States,

(2) University of Massachusetts Dartmouth, School for Marine Science and Technology, New Bedford, MA, 02744, United States

* Corresponding Author: Lin Zhang (lin.zhang@tamucc.edu)

†Present Addresses: Woods Hole Oceanographic Institution, Woods Hole, 02543, MA, United States

Contents of this file

Figure SF1 -----------------------------------P. S-2

Figure SF2 -----------------------------------P. S-3

Figure SF3 -----------------------------------P. S-4

Figure SF4 -----------------------------------P. S-5

Figure SF5 -----------------------------------P. S-5

Figure SF6 -----------------------------------P. S-6

Figure SF7 -----------------------------------P. S-6

Table ST1 -----------------------------------P. S-7

Table ST2 -----------------------------------P. S-8

Table ST3 -----------------------------------P. S-9

Text S1 -----------------------------------P. S-10

Text S2 -----------------------------------P. S-11-12

Reference -----------------------------------P. S-13

Introduction

This file contains the biosynthetic and catabolic pathways of histidine (Figure SF1), ion-exchange chromatograms of standard mixture and samples (Figure SF2), quantification of IC-collected HIS fractions (Figure SF3), the concentration of NO_3^- yielded from UV + POR oxidation versus HIS concentration (Figure SF4), linear regression of $\delta^{15}N_{UV+POR}$ versus $\delta^{15}N_{HIS\text{-}Total}$ of HIS isotopic standards (Figure SF5), linear regression of $\delta^{15}N_{ClO}$ versus $\delta^{15}N_{HIS\text{-}Total}$ of HIS isotopic standards (Figure SF6), a graphical representation of position-specific $\delta^{15}N_{HIS}$ of samples (Figure SF7), details of sample collection (Table ST1), results of UV+POR oxidation (Table ST2), results of NaClO oxidation (Table ST3), the experimental procedures of NaClO oxidation and calibration standards of $\delta^{15}N$-N_2O analysis (Text S1), and the details about the calibration and correction of $\delta^{15}N_{HIS}$ data (Text S2).

Supplementary materials

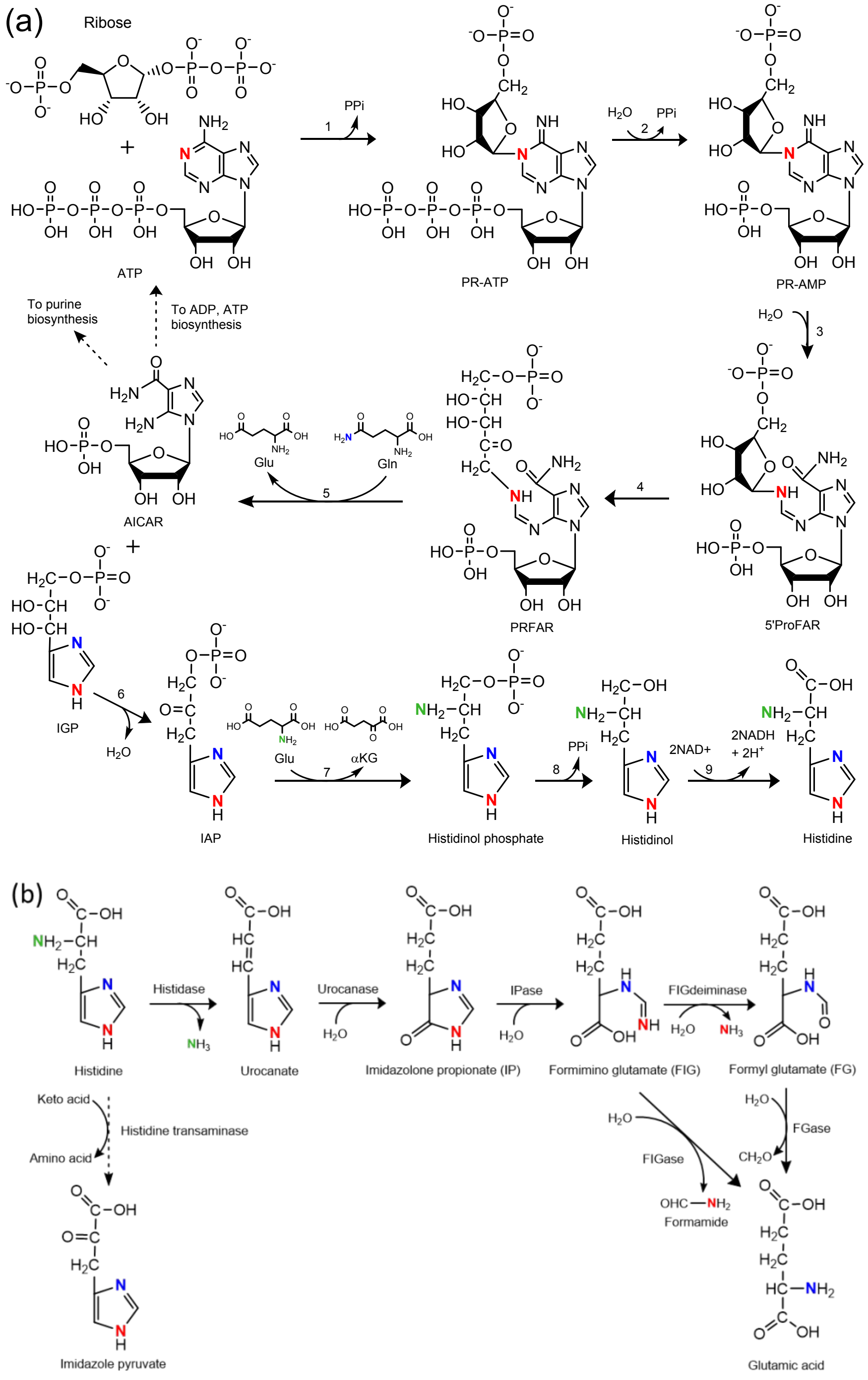

Figure SF1: (a) Biosynthetic pathway of histidine[1, 2]. The enzymes are: (1) ATP phosphoribosyl transferase; (2) phosphoribosyl-ATP pyrophosphohydrolase; (3) phosphoribosyl-AMP cyclohydrolase; (4) phosphoribosylformimino-5-aminoimidazole carboxamide ribotide isomerase; (5) imidazole glycerol-phosphate synthase; (6) imidazoleglycerol-phosphate dehydratase; (7) histidinol-phosphate aminotransferase; (8) histidinol phosphate phosphatase; (9) histidinol dehydrogenase. (b) Catabolic pathways of histidine involving the breakage of C-N bond.

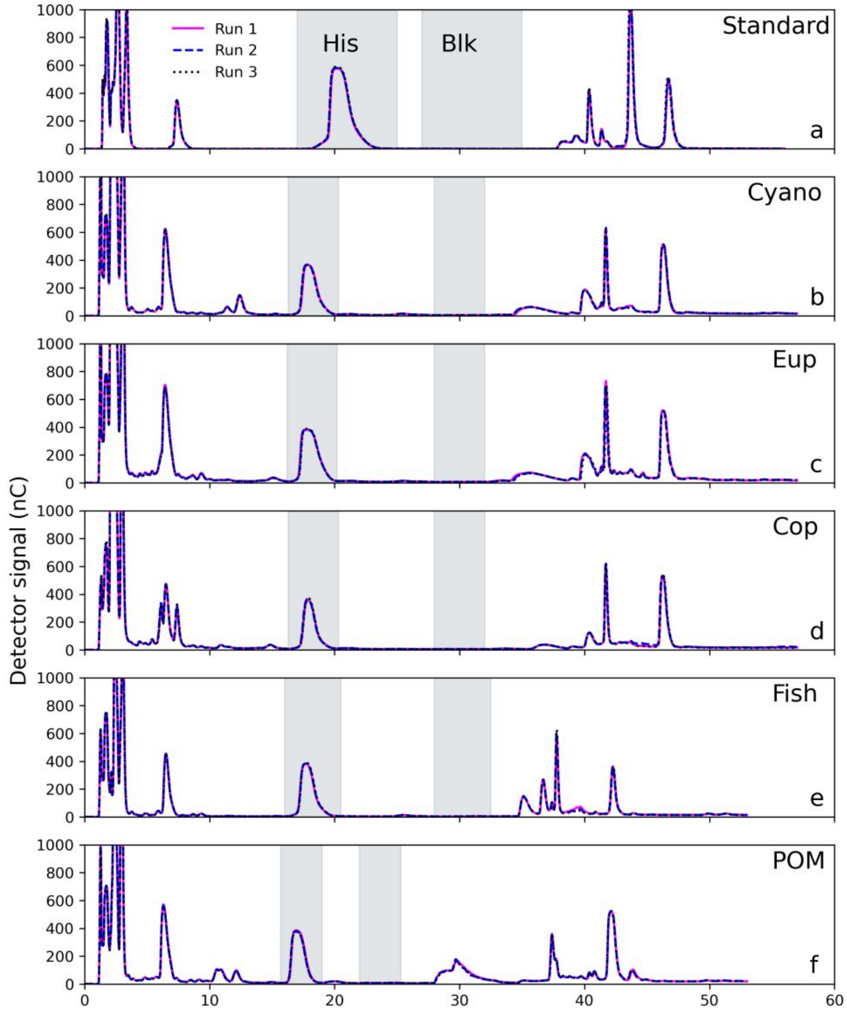

Figure SF2. IC chromatograms of (a) standard mixture; (b) cyanobacteria; (c) euphausiid; (d) copepod; (e) fish; and (f) POM. Replicate injections were overlaid. The shaded areas denote the fraction collection windows of HIS and blank.

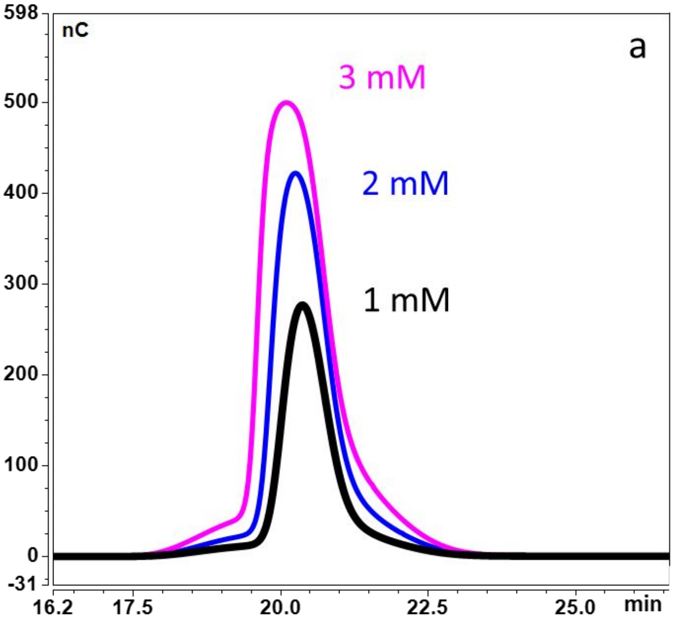

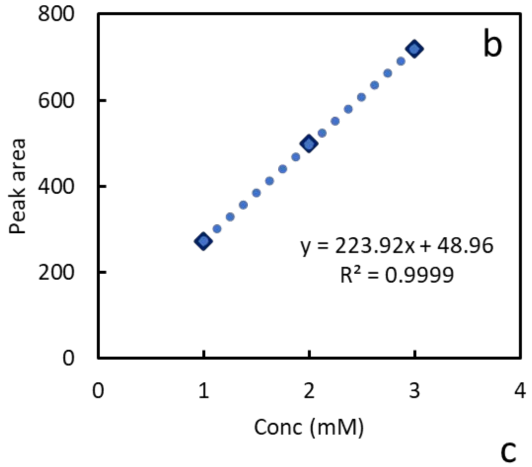

c

	Area	His Conc (mM)	Amount of His per injection (nmol)
Copepod 1	689	2.86	71.5
Copepod 2	710	2.95	73.8

Figure SF3. (a) IC chromatograms of HIS standards of 1, 2, and 3 mM. (b) Calibration curve obtained by plotting HIS peak area versus concentration. (c) An example of quantifying the concentration of HIS in replicate injections of a natural sample with the sample peak area and the calibration curve shown in (b). The amount of HIS (nmol) was calculated based on an injection volume of 25 μl.

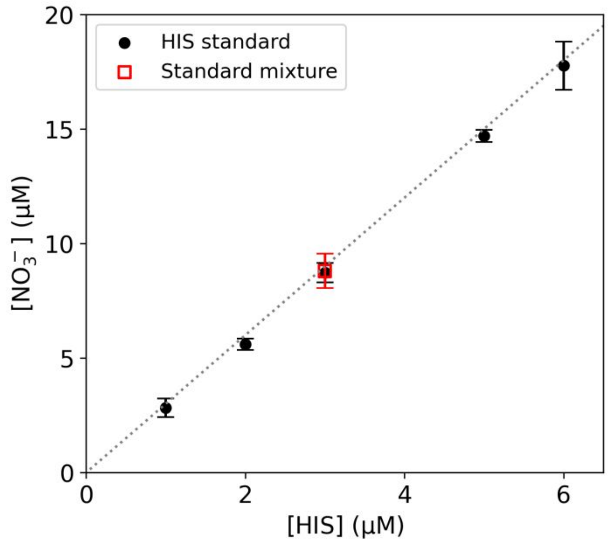

Figure SF4. The concentration of NO_3^- yielded from UV + POR oxidation on HIS isotopic standards in various concentrations and IC HIS fractions collected from injections of standard mixtures. The dotted line indicates the 100% yield with a ratio of 3:1 for $[NO_3^-]$:[HIS].

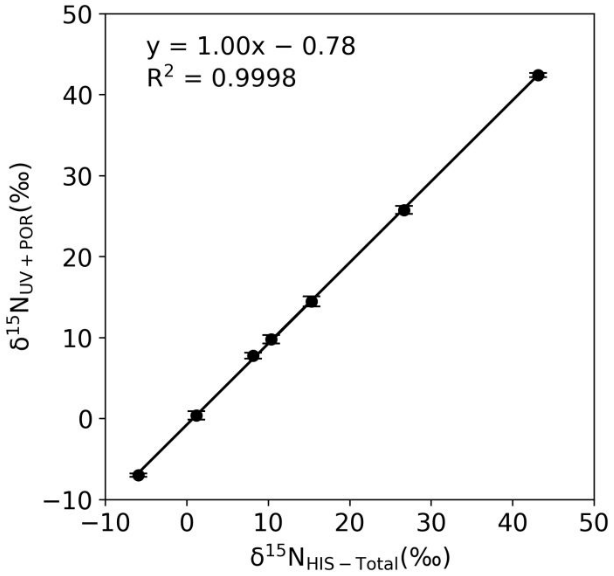

Figure SF5. Comparison of $\delta^{15}N$ values of HIS measured by two methods. $\delta^{15}N_{UV+POR}$ values represent the values obtained from UV-persulfate oxidation of HIS isotopic standards prepared in MilliQ water, while $\delta^{15}N_{HIS\text{-}Total}$ values were determined independently by EA-IRMS (Table 1). The strong linear correlation (slope ≈ 1, small y-intercepts) demonstrates excellent agreement between the two methods for determining the molecular average $\delta^{15}N$ values of HIS.

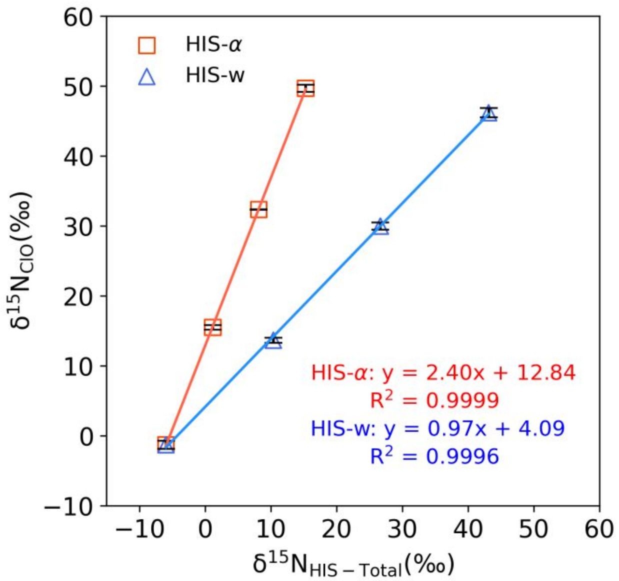

Figure SF6. $\delta^{15}N_{ClO}$ values (measured from NaClO oxidation) of HIS isotopic standards prepared in MilliQ water are plotted against their $\delta^{15}N_{HIS\text{-}Total}$ values determined by EA-IRMS (Table 1). $\delta^{15}N_{ClO}$ values reflect a weighted average of the $\delta^{15}N$ values from both the α-N and sidechain-N positions. The slope of HIS-α series was used to calculate the proportion of NO_2^- produced from α-N, according to equations (4) and (6) in the main text. The y-intercept of the HIS-α series can be expressed as: $c_{\alpha,\, ClO} = (1-3r)\,\delta^{15}N_{His\text{-}s0} + r\,\varepsilon_\alpha + (1-r)\,\varepsilon_s$, where r is the fraction of NO_2^- derived from α-N, and ε_α and ε_s are the fractionation factors for NaClO oxidation at the α- and sidechain-N positions, respectively. If NaClO oxidation were specific only to the α-N, the slope of the HIS-α series (in which only $\delta^{15}N_{HIS\text{-}\alpha}$ increases) would be 3. Observed slopes < 3 suggest a minor but measurable contribution from sidechain-N to the $\delta^{15}N_{ClO}$ values.

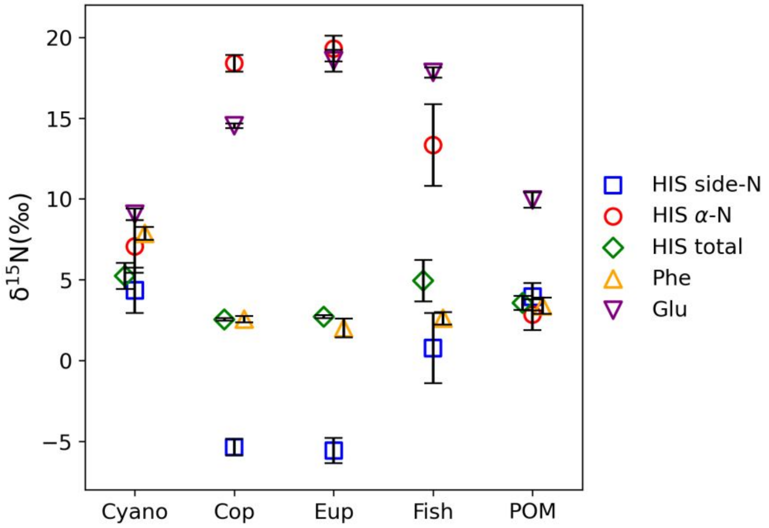

Figure SF7. $\delta^{15}N$ values of α-N and side-N of natural samples calculated from $\delta^{15}N_{UV+POR}$ values (calibrated to $\delta^{15}N_{HIS\text{-}Total}$ values) and $\delta^{15}N_{ClO}$ values of IC-collected HIS fractions. The values of $\delta^{15}N_{Phe}$ and $\delta^{15}N_{Glu}$ are included for comparison.

Sample name	Sample details	Collection location	Collection date	Reference
Cyanobacteria	*Spirulina Pacifica*, commercial powder	/	/	[3, 4]
Copepod	> 1 mm, picked from mixed tow	ETNP (14.02 N, 104.27 W). 30 m	Dec 2020	[5]
Euphausiid	> 1 mm, picked from mixed tow	ETNP (14.02 N, 104.27 W). 80 m	Dec 2020	[5]
Fish	Striped mullet muscle tissue	Nueces Bay, TX, USA	May 2014	/
POM 1	In-situ filtration of ~ 280L of seawater. 0.3 – 53 µm on GF 75 filter	ETNP (14.02 N, 104.27 W). 35 m	Dec 2020	[6]

Table ST1: Details of sample collection.

Sample	Matrix	Initial conc of total N (μM)	NO_3^- conc (μM)	Average Oxidation yield (%)
Pure standards				
HIS	MilliQ water	15.0	14.4 ± 0.7	96 ± 5
HIS	0.4 M NaOH	15.0	15.5 ± 0.8	103 ± 3
Urocanic acid	MilliQ water	16.0	14.7 ± 0.4	92 ± 3
IC HIS fractions				
Standard mixtures	0.4 M NaOH	9.0	8.8 ± 0.8	100 ± 6
Cyanobacteria	0.4 M NaOH	9.1	8.5 ± 0.8	93 ± 8
Copepod	0.4 M NaOH	8.0	6.7 ± 1.3	89 ± 10
Euphausiid	0.4 M NaOH	11.6	10.4 ± 0.4	94 ± 4
Fish	0.4 M NaOH	8.2	6.8 ± 0.1	84 ± 2
POM	0.4 M NaOH	10.3	10.7 ± 0.6	103 ± 6

Table ST2. Results of UV + POR oxidation on HIS isotopic standards and IC HIS fractions collected from injections of standard mixtures and natural samples. Oxidation yields were determined by the concentration of NO_3^- produced relative to the initial concentration of total N, averaged over triplicate samples.

Sample	Matrix	HIS or urocanic acid# conc (μM)	NO_2^- conc (μM)	Average oxidation yield (%)
Pure standards				
HIS	MilliQ water	4.8	3.8 ± 0.1	80 ± 2
Urocanic acid#	MilliQ water	4.8	0.4 ± 0.1	9 ± 1
Urocanic acid#	0.4 M NaOH	4.8	0.1 ± 0.1	3 ± 2
IC HIS fractions				
Standard AA mixtures	0.4 M NaOH	3.7	2.8 ± 0.2	75 ± 6
Cyanobacteria	0.4 M NaOH	2.4	1.8 ± 0.1	72 ± 5
Copepod	0.4 M NaOH	2.4	1.7 ± 0.2	73 ± 3
Euphausiid	0.4 M NaOH	3.1	2.8 ± 0.2	89 ± 6
Fish	0.4 M NaOH	2.8	2.1 ± 0.1	74 ± 3
POM	0.4 M NaOH	3.3	2.6 ± 0.1	80 ± 3

Table ST3. Oxidation results of NaClO reaction on HIS isotopic standards and IC HIS fractions collected from injections of standard mixtures and natural samples. Oxidation yields were calculated as the concentration of NO_2^- produced relative to the initial concentration of HIS or urocanic acid. The average oxidation yields of triplicate samples were reported.

Supporting information text S1

NaClO oxidation

The procedure of NaClO oxidation is adopted from the methods described in Strickland-Parsons[7] and Zhang & Altabet[8] based on the following mechanisms. The α-N of HIS was first converted to NH_3 by ClO^-, then quantitatively oxidized to NO_2^- by excess ClO^- under alkaline condition:

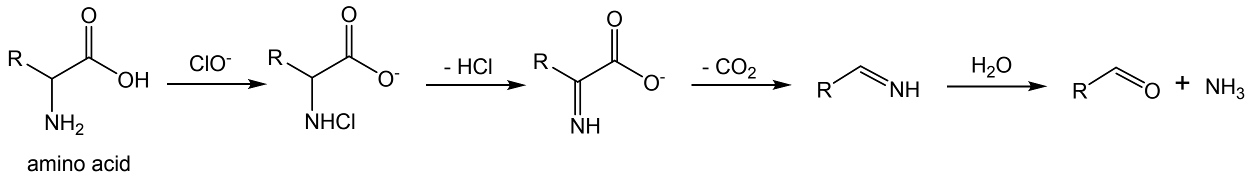

$$3ClO^- + NH_3 + OH^- \longrightarrow NO_2^- + 2H_2O + 3Cl^-$$

The catalytic reagent (0.665 g NaBr in 100 mL 10M NaOH) and oxidizing reagent (4.25 mL of 5.25% NaClO diluted to 100 mL) were freshly prepared. Each reaction vial contained 50 nmol of HIS/ urocanic acid standard or IC-collected fractions (~ 3.5 – 10 ml), to which 0.1 mL of catalytic reagent and 0.1 mL of oxidizing reagent were sequentially added, raising the pH of the samples to above 12. Under this range of pH, all the amine groups in HIS and urocanic acid would be protonated, given their pKa values (HIS α-N: 9.33; HIS sidechain-N: 6.04; urocanic acid: ~ 6-7). After incubation at 50°C for 30 minutes in a water bath, the reaction was quenched with 0.2 ml of Na_2AsO_2 solution (5.1 g of Na_2AsO_2 in 100 ml of MilliQ water).

Calibration standards of $\delta^{15}N$-N_2O analysis

NO_3^- obtained from UV+POR oxidation was reduced to N_2O in serum vials by chemical conversion using Ti (III)[9]. To calibrate the unknowns, triplicates of MilliQ water blanks, USGS34, USGS35, and an in-house NO_3^- isotopic reference standard received the same treatment as the samples. NO_2^- in samples oxidized by NaClO along with triplicates of MilliQ water blanks and three in-house NO_2^- isotopic reference standards, were converted to N_2O by NaN_3 in serum vials. $\delta^{15}N$ analysis of N_2O was conducted using a purge-and-trap continuous-flow isotope ratio mass spectrometer (PT/CF/IRMS; Isoprime Ltd). MilliQ water blanks, and NO_3^- and NO_2^- isotopic reference standards were analyzed at the beginning and end of each sample analysis sequence.

Supporting information text S2

Blank correction

Reaction blank correction was performed using an isotope mass balance equation[4, 10, 11] to obtain $\delta^{15}N$ values for the UV + persulfate oxidation ($\delta^{15}N_{UV + POR}$) and NaClO oxidation ($\delta^{15}N_{ClO}$) of HIS samples:

$$\delta^{15}N_{UV+POR\ or\ ClO} = \frac{\delta^{15}N_s\, A_s - \delta^{15}N_{blk}\, A_{blk}}{A_s - A_{blk}} \qquad (9)$$

where $\delta^{15}N_s$ and A□ represent the measured $\delta^{15}N$ and N content (in nmol) of the HIS samples, and $\delta^{15}N_{blk}$, and A_{blk} correspond to the same parameters for procedural blanks. HIS and urocanic acid standards directly oxidized by UV + POR or NaClO were corrected using the respective reaction blanks, while IC procedural blanks were used to correct HIS fractions collected from IC separations of both standard mixtures and natural samples.

Error Analysis and Propagation

Propagated error of $\delta^{15}N_{UV+POR}$ and $\delta^{15}N_{ClO}$ measurements

The propagated error of $\delta^{15}N_{UV+POR}$ and $\delta^{15}N_{ClO}$ from the blank subtraction using equation (9) can be expressed as the following suggested by Polissar et al.[11]:

$$\sigma_{\delta^{15}N_{UV+POR\ or\ ClO}}{}^2 = \left(\frac{\partial\delta^{15}N_{UV+POR\ or\ ClO}}{\partial A_s}\right)^2 \sigma_{A_s}{}^2 + \left(\frac{\partial\delta^{15}N_{UV+POR\ or\ ClO}}{\partial\delta^{15}N_s}\right)^2 \sigma_{\delta^{15}N_s}{}^2 + \left(\frac{\partial\delta^{15}N_{UV+POR\ or\ ClO}}{\partial A_{P\_Blk}}\right)^2 \sigma_{A_{P\_Blk}}{}^2 + \left(\frac{\partial\delta^{15}N_{UV+POR\ or\ ClO}}{\partial\delta^{15}N_{P\_Blk}}\right)^2 \sigma_{\delta^{15}N_{P\_Blk}}{}^2 \qquad (10)$$

For a single measurement of HIS samples, σ_{A_s} is omitted. Equation (2) becomes:

$$\sigma_{\delta^{15}N_{UV+POR\ or\ ClO}}{}^2 = \left(\frac{A_s}{A_s\text{-}A_{Blk}}\right)^2 \sigma_{\delta^{15}N_s}{}^2 + \left(\frac{\text{-}A_{Blk}}{A_s\text{-}A_{Blk}}\right)^2 \sigma_{\delta^{15}N_{Blk}}{}^2 + \left(\frac{\delta^{15}N_{UV+POR\ or\ ClO}\text{-}\delta^{15}N_{Blk}}{A_s\text{-}A_{Blk}}\right)^2 \sigma_{A_{Blk}}{}^2 \qquad (11)$$

$\sigma_{\delta^{15}N_s}$ includes the uncertainty of the isotopic measurement of the HIS samples by the IRMS, and the error propagated from the blank correction of Ti(III) or NaN_3 reaction blanks and $\delta^{15}N\text{-}NO_3^-$ or $\delta^{15}N\text{-}NO_2^-$ normalization by linear regression, which is generally below 0.2‰.

The propagated uncertainty of $\delta^{15}N_{HIS\text{-}Total}$ can then be calculated by the following based on equation (2):

$$\sigma_{\delta^{15}N_{HIS\text{-}Total}} = \sqrt{\left[\frac{\sigma_{\delta^{15}N_{UV+POR}}{}^2 + \sigma_c{}^2}{(\delta^{15}N_{UV+POR}\text{-}c)^2} + \left(\frac{\sigma_m}{m}\right)^2\right] \cdot \delta^{15}N_{HIS\text{-}Total}{}^2} \qquad (12)$$

Where σ_m and σ_c are the uncertainties of the slope and intercept of $\delta^{15}N_{UV+POR}$ versus $\delta^{15}N_{HIS\text{-}Total}$ of HIS isotopic standards or IC HIS fractions of standard mixtures.

Propagated error of $\delta^{15}N_{HIS\text{-}\alpha}$ and $\delta^{15}N_{HIS\text{-}s}$ calculations

$\delta^{15}N_{HIS\text{-}s}$ were calculated based on equation (5), which can be simplified as the following:

$$\delta^{15}N_{His\text{-}s} = \frac{m_{\alpha,ClO}\ \delta^{15}N_{HIS\text{-}Total} - \delta^{15}N_{ClO} + \varepsilon}{m_{\alpha,ClO} - 1}, \ \varepsilon = r\varepsilon_\alpha + (1-r)\,\varepsilon_s$$

The propagated error of $\delta^{15}N_{HIS\text{-}s}$ could be expressed as the following:

$$\sigma_{\delta^{15}N_{HIS\text{-}s}}{}^2=\left(\frac{\partial\delta^{15}N_{HIS\text{-}s}}{\partial m_{\alpha,ClO}}\right)^2\sigma_{m_{\alpha,ClO}}{}^2+\left(\frac{\partial\delta^{15}N_{HIS\text{-}s}}{\partial\delta^{15}N_{HIS\text{-}Total}}\right)^2\sigma_{\delta^{15}N_{HIS\text{-}Total}}{}^2+\left(\frac{\partial\delta^{15}N_{HIS\text{-}s}}{\partial\delta^{15}N_{ClO}}\right)^2\sigma_{\delta^{15}N_{ClO}}{}^2+\left(\frac{\partial\delta^{15}N_{HIS\text{-}s}}{\partial\varepsilon}\right)^2\sigma_{\varepsilon}{}^2$$

$$\sigma_{\delta^{15}N_{HIS\text{-}s}}{}^2=\left(\frac{\delta^{15}N_{HIS\text{-}Total}-\delta^{15}N_{ClO}+\varepsilon}{(m\text{-}1)^2}\right)^2\sigma_{m_{\alpha,ClO}}{}^2+\left(\frac{m}{m\text{-}1}\right)^2\sigma_{\delta^{15}N_{HIS\text{-}Total}}{}^2+\left(\frac{1}{1\text{-}m}\right)^2\sigma_{\delta^{15}N_{ClO}}{}^2+\left(\frac{1}{m\text{-}1}\right)^2\sigma_{\varepsilon}{}^2 \quad (13)$$

σ_ε could be calculated by simple error propagation rules based on the uncertainty of ε_α, ε_s, and r.

With $\sigma_{\delta^{15}N_{HIS\text{-}s}}$, the propagated error of $\delta^{15}N_{HIS\text{-}\alpha}$ was then calculated by equation (14):

$$\delta^{15}N_{His-\alpha} = 3\,\delta^{15}N_{HIS-Total} - 2\,\delta^{15}N_{His-s}$$

$$\sigma_{\delta^{15}N_{His\text{-}\alpha}}=\sqrt{(\sigma_{\delta^{15}N_{HIS\text{-}Total}}{}^2+\sigma_{\delta^{15}N_{HIS\text{-}s}}{}^2)} \quad (14)$$

References

(1) Alifano, P.; Fani, R.; Liò, P.; Lazcano, A.; Bazzicalupo, M.; Carlomagno, M. S.; Bruni, C. B. Histidine biosynthetic pathway and genes: structure, regulation, and evolution. *Microbiological Reviews* **1996**, *60* (1), 44-69. DOI: 10.1128/mr.60.1.44-69.1996 From NLM.

(2) Stepansky, A.; Leustek, T. Histidine biosynthesis in plants. *Amino Acids* **2006**, *30* (2), 127-142. DOI: 10.1007/s00726-005-0247-0.

(3) Broek, T. A. B.; McCarthy, M. D. A new approach to δ15N compound-specific amino acid trophic position measurements: preparative high pressure liquid chromatography technique for purifying underivatized amino acids for stable isotope analysis. *Limnol. Oceanogr. Methods* **2014**, *12* (12), 840-852. DOI: https://doi.org/10.4319/lom.2014.12.840 (accessed 2025/03/14).

(4) Zhang, L.; Lee, W.-m.; Kreider-Mueller, A.; Kuhnel, E.; Baca, J.; Ji, C.; Altabet, M. High-precision measurement of phenylalanine and glutamic acid δ15N by coupling ion-exchange chromatography and purge-and-trap continuous-flow isotope ratio mass spectrometry. *Rapid Commun. Mass Spectrom.* **2021**, *35* (13), e9085. DOI: https://doi.org/10.1002/rcm.9085.

(5) Sánchez-Velasco, L.; García-De León, F. J.; Ruvalcada-Aroche, E. D.; Beier, E.; Godínez, V. M.; Jiménez-Rosenberg, S. P. A.; Sánchez-Pérez, E. D.; Contreras-Catala, F.; Mnich, A.; Verma, N.; et al. Vertical distribution of zooplankton groups, with an emphasis on fish larvae, in the oxygen minimum zone off southern México (December 2020). *Journal of Marine Systems* **2022**, *236*, 103801. DOI: https://doi.org/10.1016/j.jmarsys.2022.103801.

(6) Lee, C. W. M.; Altabet, M.; Mnich, A.; Zhang, L. Using δ15N of Amino Acids and Nitrate to Investigate Particle Production and Transformation in the Ocean: A Case Study From the Eastern Tropical North Pacific Oxygen Deficient Zone. *Global Biogeochem. Cycles* **2025**, *39* (1), e2024GB008280. DOI: https://doi.org/10.1029/2024GB008280 (accessed 2025/03/14).

(7) Strickland, J. D. H.; Parsons, T. R. *A practical handbook of seawater analysis*; Fisheries Research Board of Canada, 1972. DOI: http://dx.doi.org/10.25607/OBP-1791.

(8) Zhang, L.; Altabet, M. A. Amino-group-specific natural abundance nitrogen isotope ratio analysis in amino acids. *Rapid Commun. Mass Spectrom.* **2008**, *22* (4), 559-566. DOI: https://doi.org/10.1002/rcm.3393.

(9) Altabet, M. A.; Wassenaar, L. I.; Douence, C.; Roy, R. A Ti(III) reduction method for one-step conversion of seawater and freshwater nitrate into N2O for stable isotopic analysis of 15N/14N, 18O/16O and 17O/16O. *Rapid Commun. Mass Spectrom.* **2019**, *33* (15), 1227-1239. DOI: https://doi.org/10.1002/rcm.8454.

(10) McIlvin, M. R.; Altabet, M. A. Chemical Conversion of Nitrate and Nitrite to Nitrous Oxide for Nitrogen and Oxygen Isotopic Analysis in Freshwater and Seawater. *Anal. Chem.* **2005**, *77* (17), 5589-5595. DOI: https://10.1021/ac050528s.

(11) Polissar, P. J.; Fulton, J. M.; Junium, C. K.; Turich, C. C.; Freeman, K. H. Measurement of 13C and 15N Isotopic Composition on Nanomolar Quantities of C and N. *Anal. Chem.* **2009**, *81* (2), 755-763. DOI: 10.1021/ac801370c.
